# *Plant Methods *moves to fund open-access publishing

**DOI:** 10.1186/1746-4811-2-6

**Published:** 2006-03-03

**Authors:** Brian G Forde, Michael R Roberts

**Affiliations:** 1 Editor-in-Chief, *Plant Methods*, Dept. of Biological Sciences, Lancaster University, Lancaster, LA1 4YQ, UK; 2 Deputy Editor, *Plant Methods*, Dept. of Biological Sciences, Lancaster University, Lancaster, LA1 4YQ, UK

## Abstract

As an Open Access journal dedicated to promoting technological innovation in plant biology, *Plant Methods *occupies a unique niche amongst plant journals. To fund its open access policy, and to enable it to continue to serve the plant sciences community, *Plant Methods *will be introducing an article processing charge (APC) from March 1^st ^2006. This charge will cover the costs of making the article freely and universally accessible online and the costs involved in its inclusion in PubMed and its archiving in public repositories. In some circumstances, waivers of the APC may be granted and authors whose institutions are BioMed Central members will incur no, or reduced, charges.

## Introduction

*Plant Methods *is breaking new ground in plant science publishing by providing an open access outlet for refereed articles specifically devoted to technological innovation in plant biology. *Plant Methods *is published by BioMed Central and all its content is universally and freely available online to everyone, its authors retain copyright, and it is archived in PubMed Central [1].

The traditional model for scientific publishing is that readers pay to access articles, either through subscriptions or by paying a fee each time they download an article. These access charges inevitably limit how many will be able to read, use and cite the articles. Furthermore, while the perception may be that traditional journals do not charge authors for publication, it is common for them to levy a fee for colour figures – and many additionally impose page charges.

## Why open access?

Open access changes the way in which articles are published. First, all articles become freely and universally accessible online, and so an author's work can be read by anyone at no cost. Second, the authors hold copyright for their work and grant anyone the right to reproduce and disseminate the article, provided that it is correctly cited and no errors are introduced [1]. Third, a copy of the full text of each article is permanently archived in an online repository separate from the journal. *Plant Methods' *articles are archived in PubMed Central [3], the US National Library of Medicine's full-text repository of life science literature, and also in repositories at the University of Potsdam [4] in Germany, at INIST [5] in France and in e-Depot [6], the National Library of the Netherlands' digital archive of all electronic publications.

Open access has four broad benefits for science and the general public.

• First, authors are assured that their work is disseminated to the widest possible audience, given that there are no barriers to access their work. This is accentuated by the authors being free to reproduce and distribute their work, for example by placing it on their institution's website. Open access journals reach a much larger set of readers than any subscription-based journal, in print and online [7]. Some studies have suggested a correlation between open access, higher downloads and higher citations, leading to a higher Impact Factor [8].

• Second, the information available to readers will not be limited by their library's budget, and the widespread availability of articles will enhance literature searching [9].

• Third, the results of publicly funded research will be accessible to all taxpayers and not just those with access to a library with a subscription. As such, open access could help to increase public interest in, and support of, research.

• Fourth, a country's economy will not influence its researchers' ability to access articles because resource-poor countries (and institutions) will be able to read the same material as wealthier ones.

## The APC payment

To fund its open access policy, authors of articles accepted for publication in *Plant Methods *will be asked to pay an article-processing charge (APC) of £750. This will apply to articles submitted from March 1^st ^2006. These APCs will allow continued open access to all of *Plant Methods' *articles. Waiver requests will be considered by the Editor-in-Chief on a case-by-case basis. If an author is affiliated to a BMC member institute, the institute will cover all or a portion of the APC. Current member institutions are listed here . No charge is made for articles that are rejected after peer review. Many funding agencies have also realized the importance of open access publishing and have specified that their grants may be used directly to pay APCs .

## What the APC covers

The APC pays for efficient and thorough peer review, for the article to be freely and universally accessible in various formats online, and for the processes required for inclusion in PubMed and archiving in PubMed Central, e-Depot, Potsdam and INIST. Although £750 may seem expensive, it must be remembered that (unlike other journals) *Plant Methods *will not levy additional page or colour charges, which in other journals can easily exceed £750. Indeed, with the article being published online only, any number of colour figures and photographs can be included, at no extra cost.

## Free access versus open access

Although several journals now offer free access to their articles online, this is different from open access (as defined by the Bethesda Statement [10]). Journals often delay free access for 6–12 months, and even when the full text is available, readers are not allowed to reproduce and/or disseminate the work because of restrictions imposed by the copyright policy. That said, *Plant Methods *and other BioMed Central journals are not alone in the move to open access funded by APCs. For example, the Public Library of Science has set up new open access journals, and have elected to set APCs of US$1500 for each accepted article [11]. Given that the Public Library of Science has used television advertising to promote journals [12], the high profile of these journals will raise awareness of open access and encourage researchers in all disciplines to understand and accept open access, with APCs as an acceptable method to fund it.

## Conclusion

It is only through the instigation of article processing charges that *Plant Methods *will be able to continue serving the community of plant biologists by promoting technological innovation in the plant sciences. If you have recently devised a novel protocol or research tool that you think may be of value to other plant biologists, we invite you to support this exciting new initiative by submitting your paper to *Plant Methods.*

## Abbreviations

APC = article-processing charge.
